# Pharmacokinetic study on the interaction between pachymic acid and bavachin and its potential mechanism

**DOI:** 10.1080/13880209.2021.1942924

**Published:** 2021-09-13

**Authors:** Jie Zhang, Lu Liu, Hong Li, Bin Zhang

**Affiliations:** a Department of Medicinal Medicine, The Second Hospital of Shandong University, Jinan, China; b Department of Endocrinology, Seventh People's Hospital of Shanghai University of TCM, Shanghai, China;; c Department of Endocrinology and Metabolism, Shanghai Tenth People's Hospital, Tongji University School of Medicine, Shanghai, China

**Keywords:** CYP2C9, *P-gp*, drug-drug interaction

## Abstract

**Context:**

Pachymic acid and bavachin are commonly used drugs in the therapy of lung cancer.

**Objective:**

The co-administration of pachymic acid and bavachin was investigated to evaluate their potential drug-drug interaction.

**Materials and methods:**

The pharmacokinetics of bavachin (10 mg/kg) was studied in male Sprague-Dawley (SD) rats in the presence of pachymic acid (5 mg/kg) (*n* = 6). The rats without pre-treatment of pachymic acid were set as the control and the pre-treatment of pachymic acid was conducted for 7 days before the administration of bavachin. The effect of pachymic acid on the activity of CYP2C9 was also estimated in rat liver microsomes with corresponding probe substrates.

**Results:**

Pachymic acid influenced the pharmacokinetic profile of bavachin with the increased *AUC* (32.82 ± 4.61 *vs.* 19.43 ± 3.26 μg/L/h), the prolonged *t*_1/2_ (3.21 ± 0.65 *vs.* 2.32 ± 0.28 h), and the decreased *CLz*/*F* (307.25 ± 44.35 *vs.* 523.81 ± 88.67 L/h/kg) *in vivo*. The metabolic stability of bavachin was enhanced by pachymic acid and the transport of bavachin was inhibited by pachymic acid. Pachymic acid was found to inhibit the activity of CYP2C9 with the IC_50_ of 21.25 µM as well as the activity of *P-gp*.

**Discussion and conclusion:**

The interaction between pachymic acid and bavachin results from the inhibition of CYP2C9 and *P-gp*. The dose of bavachin should be adjusted when combining with pachymic acid. The study design can be generalized to a broader study population with adjustment in the dose.

## Introduction

In the clinic, traditional Chinese medicine has been considered as an important source for the anticancer agent and has been currently used in practice (Feng et al. 2011). *Poria cocos* (Schw.) Wolf (Polyporaceae) is a well-known traditional Chinese medicine, with a variety of pharmacological effects (Zhang et al. 2018). Pachymic acid is one of the major natural compounds of *P. cocos*, which has been demonstrated to possess the effects of immunomodulatory, antitumor, anti-inflammation, and antioxidation (Kim et al. 2013; Lee et al. 2013; Zhang et al. 2017). Previous studies have reported that pachymic acid could inhibit cell growth and induce cell apoptosis of lung cancer, which makes it frequently applied in the clinical prescription for lung cancer (Ling et al. 2010; Ma et al. 2015). In the therapy of lung cancer, *Psoralea corylifolia* Linn. (Fabaceae) is also a commonly used herbal medicine, which can inhibit cell activity of lung cancer (Yin et al. 2019). Bavachin is the main flavonoid in *P. corylifolia* that has been reported to have antibacterial, anti-inflammation, and antidiabetic activities (Yin et al. 2004; Alam et al. 2018; Hung et al. 2019).

Low or no response or resistance to the therapeutic drug is the general reason for the failure in the clinical treatment (Lin and Shaw 2016). Co-administration of different types of drugs is a common preventable cause of these potential adverse effects (Dechanont et al. 2014). Previously, Balap et al. (2017) reported that co-administration of *Andrographis paniculate* (Burm. F) Nees (Acanthaceae) extract and pure andrographolide with naproxen decreased the systemic exposure level of naproxen and suppressed its anti-arthritic activity. Pachymic acid and bavachin could be used together in the clinical treatment of lung cancer, which may induce adverse interaction and even toxicity. Therefore, *in vivo* investigation on the co-administration of pachymic acid and bavachin is of great necessity to guide the clinical combination of different types of herbs or drugs.

Here, pachymic acid was co-administrated with bavachin in rats to investigate the interaction between these two herbs, and its potential mechanism was also studied.

## Materials and methods

### Chemicals

Pachymic acid and bavachin were purchased from Shanghai Standard Biotechnology Co., Ltd (Shanghai, China). Acetonitrile and methanol were purchased from Fisher Scientific (Fair Lawn, NJ, USA). Dulbecco’s modified Eagle’s medium (DMEM) and non-essential amino acid (NEAA) solution were purchased from Thermo Scientific Corp. (Logan, UT, USA). Foetal bovine serum (FBS) was obtained from GIBCO BRL (Grand Island, NY, USA). Penicillin G (10,000 U/mL) and streptomycin (10 mg/mL) were purchased from Amresco (Solon, OH, USA). Hanks' balanced salt solution (HBSS) was purchased from GIBCO (Grand Island, NY, USA). Ultrapure water was prepared with a Milli-Q water purification system (Millipore, Billerica, MA, USA). All other chemicals were of analytical grade or better.

### Animals

This study was approved by the Animal Care and Use Committee of The Second Hospital of Shandong University. Male Sprague-Dawley (SD) rats were obtained from the Shanghai Laboratory Animal Centre, Chinese Academy of Science (Shanghai, China). All experimental animals were housed at 25 °C with 60 ± 5% humidity and a 12 h dark/light cycle. Before the experiments, rats have fasted overnight.

### Effect of pachymic acid on the pharmacokinetics of bavachin

The experimental rats were divided into two groups: the bavachin group and the co-administration group. Pachymic acid and bavachin were orally administrated to rats at doses of 5 and 10 mg/kg, respectively, according to previous studies (Cai et al. 2017; Li et al. 2017; Yang et al. 2018). For the co-administration group, the rats were pre-treated with pachymic acid for 7 days followed by the administration of bavachin to avoid the chemical reaction between these two drugs. The plasma samples (150 μL) were collected from the fossa orbitalis vein into a EDTA pre-treated centrifuge tubes after 0, 0.083, 0.25, 0.5, 1.0, 2.0, 4.0, 6.0, 8.0, 10, 12, and 24 h of the bavachin administration. The concentration of bavachin in the plasma samples was analyzed by the LC-MS/MS after centrifugation.

### LC-MS/MS analysis

The chromatographic analysis was performed with the Agilent 1290 series liquid chromatography system and an Agilent 6470 triple-quadruple mass spectrometer (Palo Alto, CA, USA) with the Waters X-Bridge C18 column (3.0 × 100 mm, i.d.; 3.5 μm, USA). The mobile phase was water (containing 0.1% formic acid) and acetonitrile (30:70, v: v) with isocratic elution at a flow rate of 0.2 mL/min, and the analysis time was 4 min.

The quantification was conducted in a multiple reaction monitoring (MRM) mode with the m/z of 323.1 → 119.0 for bavachin and 255.1 → 119.0 for liquiritigenin as the internal standard (IS). The collision energy for bavachin and IS were 30 and 20 eV, respectively. The MS/MS conditions were optimized as follows: fragmentor, 110 V; capillary voltage, 3.5 kV; Nozzle voltage, 500 V; nebulizer gas pressure (N_2_), 40 psig; drying gas flow (N_2_), 10 L/min; gas temperature, 350 °C; sheath gas temperature, 400 °C; sheath gas flow, 11 L/min.

### Effect of pachymic acid on the transport of bavachin

The transport of bavachin was investigated in the Caco-2 cell transwell model. The Caco-2 cells were obtained from ATCC (Manassas, VA, USA) and cultured in the DMEM high glucose medium with 15% FBS, 1% NEAA, and 100 U/mL penicillin and streptomycin. Cells were seeded on the transwell polycarbonate insert filters at a density of 1 × 10^5^ cells/cm^2^ and grew for 21 d. The culture medium was replaced every two days in the first seven days and then daily. The integrity of the Caco-2 monolayers was confirmed by the paracellular flux of Lucifer yellow, which was <1% per hour. The alkaline phosphatase activity was validated using an Alkaline Phosphatase Assay Kit. The qualified monolayers were used for transport studies.

The cell monolayers were washed with Hanks’ balanced salt solution twice and incubated at 37 °C for 20 min. Then, the cell monolayers were incubated with bavachin in a fresh incubation medium added on either the apical or basolateral side for the indicated times at 37 °C. A 100 μL aliquot of the incubation solution was withdrawn at the indicated time points from the receiver compartment and replaced with the same volume of fresh pre-warmed HBSS buffer. The inhibitory effects of *P-gp* inhibitors on the flux of bavachin by Caco-2 cells were investigated by adding 50 μM pachymic acid to both sides of the cell monolayers and preincubating the sample at 37 °C for 30 min. The permeability of bavachin (2 μM) in all of the above conditions for both directions, i.e., from the apical (AP) side to the basolateral (BL) side and from the BL side to the AP side, was measured after incubation for 30, 60, 90, and 120 min at 37 °C. In addition, the efflux activity of *P-gp* was validated using typical *P-gp* substrate digoxin (25 μM).

The apparent permeability coefficient (*P_app_*) was calculated using the equation of Artursson and Karlsson:

$$P_{\mathrm{app}}=(\Delta Q/\Delta t)\times [1/(A\times C_{0})]$$

where *P*_app_ is the apparent permeability coefficient (cm/s), Δ*Q*/Δ*t* (μmol/s) is the rate at which the compound appears in the receiver chamber, *C*_0_ (μmol/L) is the initial concentration of the compound in the donor chamber and *A* (cm^2^) represents the surface area of the cell monolayer. Data were collected from three separate experiments, and each was performed in triplicate.

### Effect of pachymic acid on the metabolic stability of bavachin in rat liver microsomes

Bavachin was incubated with rat liver microsomes (20 mg/mL) and PBS buffer in the centrifuge tubes on ice. The reaction was initiated by adding an NADPH-generating system after preincubation of 5 min. The effect of pachymic acid on the metabolic stability of bavachin was investigated with the preincubation of 10 μM pachymic acid at 37 °C for 30 min. The reaction was terminated by the addition of ice-cold acetonitrile containing esculin at 0.083, 0.167, 0.33, 0.5, 1, 2, 4, 8, 12, and 24 h. All the experiments were performed in triplicate. The subsequent sample preparation method was the same as the plasma sample preparation method, and the concentration of bavachin was determined by LC-MS/MS.

The *in vitro half-life* (*t*_1/2_) was obtained using the equation: *t*_1/2_ = 0.693/k; V (μL/mg) = volume of incubation (μL)/protein in the incubation (mg); Intrinsic clearance (Clint) (μL/min/mg protein) = *V* × 0.693/*t*_1/2_.

### Effect of pachymic acid on the activity of CYP2C9 enzyme

Further, the effect of pachymic acid on the enzyme activity of CYP2C9, an important enzyme responsible for the metabolism of bavachin, was investigated in rat liver microsomes. The microsomes protein was 0.5 mg/mL mixed with 50 μM diclofenac, a typical substrate of CYP2C9, and 0, 2.5, 5, 10, 25, 50, and 100 μM pachymic acid. After incubating for 30 min, the incubation was terminated by the addition of methanol, and the contents were centrifuged for further analysis by LC-MS/MS.

### Statistical analysis

The pharmacokinetic parameters were calculated by the DAS 3.0 pharmacokinetic software (Chinese Pharmacological Association, Anhui, China). The differences between groups were evaluated by one-way ANOVA with the help of GraphPad 7.0 or SPSS 23.0. *p* < 0.05 was considered statistically significant.

## Results

### Effect of pachymic acid on the pharmacokinetics of bavachin

**Figure 1.**
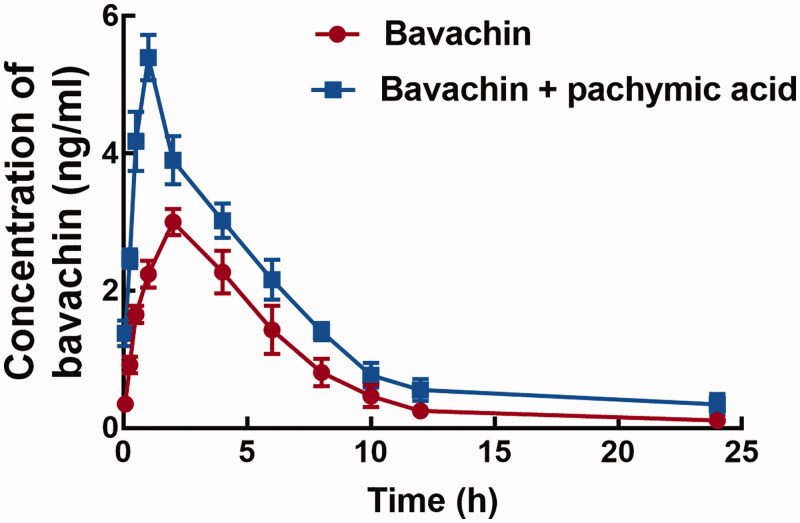
The mean plasma concentration-time curve of bavachin with or without the pre-treatment of pachymic acid. Pachymic acid significantly influenced the pharmacokinetic profile of bavachin.

The co-administration of pachymic acid significantly changed the pharmacokinetic profile of bavachin (Figure 1). The corresponding pharmacokinetic parameters were summarized in Table 1. The *AUC* of bavachin increased from 19.43 ± 3.26 to 32.82 ± 4.61 μg/L/h in the presence of pachymic acid, and the difference was statistically significant (*p* < 0.05). Consistently, the *C*_max_ (5.40 ± 0.33 *vs.* 3.03 ± 0.19 μg/L) and *t*_1/2_ (3.21 ± 0.65 *vs.* 2.32 ± 0.28 h) of bavachin also increased with the pre-treatment of pachymic acid (*p* < 0.05). Whereas, the *CLz/F* of bavachin *in vivo* was suppressed by the co-administration of pachymic acid, which decreased from 523.81 ± 88.67 to 307.25 ± 44.35 L/h/kg (*p* < 0.05).

**Table 1.** Pharmacokinetic parameters of bavachin with or without the presence of pachymic acid.

	Bavachin	Bavachin + pachymic acid
AUC_(0–_*_t_*_)_ (μg/L*h)	19.43 ± 3.26	32.82 ± 4.61*
*t*_1/2_ (h)	2.32 ± 0.28	3.21 ± 0.65*
*T*_max_ (h)	1.78 ± 0.23	0.97 ± 0.11*
*C*_max_ (μg/L)	3.03 ± 0.19	5.40 ± 0.33*
*CLz*/*F* (L/h/kg)	523.81 ± 88.67	307.25 ± 44.35*

*p < 0.05.

### Effect of pachymic acid on the transport of bavachin in Caco-2 model

**Figure 2.**
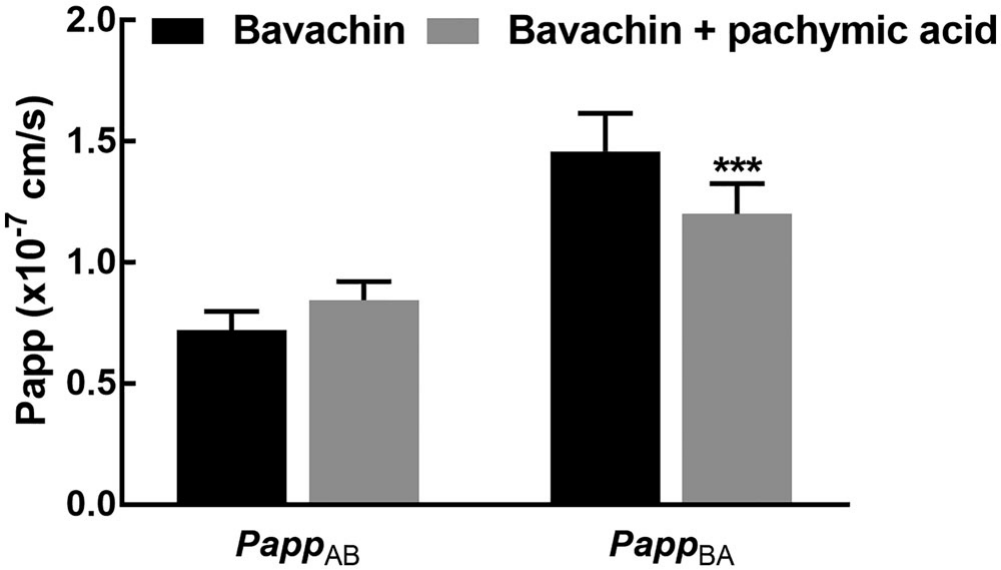
Effect of pachymic acid on the transport of bavachin in Caco-2 model. Pachymic acid significantly inhibited the efflux of bavachin due to the decrease in the value of *Papp_BA_*. ***p < 0.001.

The efflux ratio of digoxin was first investigated in the Caco-2 model to assess the activity of *P-gp*. It was found that the efflux ratio of digoxin was 12.8 in the absence of pachymic acid indicating that *P-gp* was qualified for the experiments. While the co-administration of pachymic acid inhibited the efflux ratio of bavachin to 1.21, indicating the inhibitory effect of pachymic acid on the activity of *P-gp*. The *Papp_AB_* and *Papp_BA_* of bavachin were obtained as 0.72 ± 0.08 × 10^−7^ and 1.46 ± 0.16 × 10^−7 ^cm/s, respectively. The efflux ratio was calculated as 2.06 ± 0.45, indicating the involvement of *P-gp* in the transport of bavachin. While, in the presence of pachymic acid, the *Papp_BA_* of bavachin significantly reduced to 1.20 ± 0.13 × 10^−7 ^cm/s and the efflux ratio also decreased to 1.42 ± 0.05 (Figure 2).

### Effect of pachymic acid on the metabolic stability of bavachin in rat liver microsomes

The half-life of bavachin in rat liver microsomes was 35.62 ± 3.23 min, which was prolonged to 42.75 ± 2.76 min in the presence of pachymic acid. While the intrinsic clearance rate of bavachin was also affected by the co-administration of pachymic acid. The intrinsic clearance rate of bavachin was 38.91 ± 2.15 µL/min/mg protein and it was decreased to 32.42 ± 3.43 µL/min/mg protein in the presence of pachymic acid, indicating the enhanced metabolic stability of bavachin by pachymic acid.

### Effect of pachymic acid on the activity of CYP2C9

**Figure 3.**
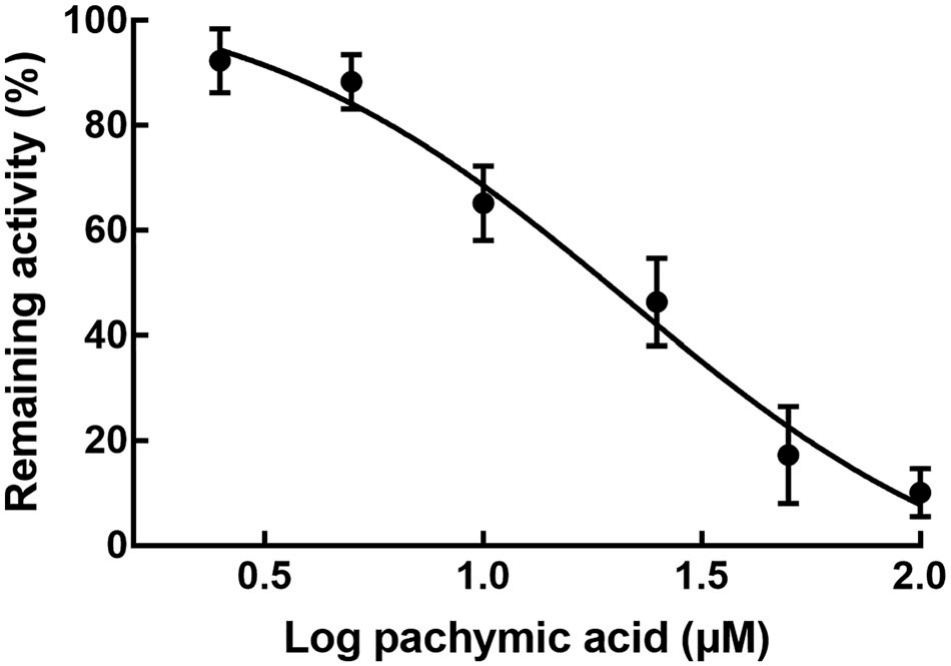
Effect of pachymic acid on the activity of CYP2C9. Pachymic acid inhibited the activity of CYP2C9 in a dose-dependent manner with the IC_50_ value of 21.25 µM.

Pachymic acid was found to inhibit the activity of CYP2C9 in rat liver microsomes. The activity of CYP2C9 was decreased with the increased concentration of pachymic acid (Figure 3). The IC_50_ value of CYP2C9 was obtained as 21.25 µM.

## Discussion

Traditional Chinese medicine is a complex mixture of different kinds of herbs, which has gained increased attention all over the world (Harvey 2008). Co-administration of various drugs possesses multi-target and multi-level function characteristics, especially in intractable diseases with complications (Huang et al. 2018). Pachymic acid and bavachin are major active ingredients of *P. cocos* and *P. corylifolia*, respectively, which are commonly combined in the therapeutic prescription of lung cancer due to their similar anti-tumor effect (Ling et al. 2010; Ma et al. 2015; Yin et al. 2019). Combination therapy of different drugs might induce adverse interaction that leads to treatment failure and even toxicity (Hu et al. 2005). Therefore, the *in vivo* interaction between co-administrated drugs should be paid special attention to evaluate the risk of the drug combination.

In the present study, it was found that pachymic acid increased the *t*_1/2_ of bavachin and decreased its clearance in rats through the pharmacokinetic study. The *in vitro* metabolic stability in rat liver microsomes was also enhanced by the co-administration of bavachin, which is consistent with the *in vivo* results. Previous reported drug-drug interaction indicated that the activity of cytochrome P450 enzymes is an important factor that mediated pharmacokinetic interaction between different drugs. For example, *Ginkgo* leaf tablets increased the plasma concentration of losartan and decreased the concentration of its metabolite via inhibiting the activity of CYP3A4 (Dong et al. 2018). Similarly, in the co-administration of berberine and losartan, berberine inhibited the activity of CYP3A4 or CYP2C9, which resulted in the inhibition in the pharmacokinetics of losartan (Li et al. 2016). In the previous study, pachymic acid was demonstrated to be a competitive inhibitor of CYP2C9, which is mainly responsible for the metabolism of bavachin (Ding et al. 2020; Li et al. 2020). Here, we also revealed the inhibitory effect of pachymic acid on the activity of CYP2C9 in a dose-dependent manner with the IC_50_ value of 21.25 µM. Therefore, it was speculated that the pharmacokinetic interaction between pachymic acid and bavachin might be a result of the inhibition of CYP2C9 by pachymic acid.

On the other hand, the activity of the transporter that participates in the absorption of drugs in the liver or intestine also plays vital roles in the pharmacokinetics of drugs and induced adverse effects (Zhao et al. 2019). The transport of bavachin was also influenced by the co-administration of pachymic acid, of which the efflux ratio significantly decreased in the presence of pachymic acid, suggesting the involvement of *P-gp* during the drug-drug interaction between pachymic acid and bavachin.

## Conclusions

Pachymic acid increased the systemic exposure of bavachin due to its inhibitory effect on the metabolism and transport of bavachin, through inhibiting the activity of CYP2C9 and *P-gp*. These results demonstrated the drug-drug interaction between pachymic acid and bavachin occurred during their co-administration. Therefore, the clinical dosage of bavachin should be adjusted, when combined with pachymic acid.
